# Neurofeedback as a Treatment for Impulsivity in a Forensic Psychiatric Population With Substance Use Disorder: Study Protocol of a Randomized Controlled Trial Combined With an N-of-1 Clinical Trial

**DOI:** 10.2196/resprot.6907

**Published:** 2017-01-25

**Authors:** Sandra Fielenbach, Franc CL Donkers, Marinus Spreen, Stefan Bogaerts

**Affiliations:** ^1^ Research Department Forensic Psychiatric Centre Dr S van Mesdag Groningen Netherlands; ^2^ Department of Developmental Psychology Tilburg University Tilburg Netherlands; ^3^ Department of Cognitive Neuroscience Maastricht University Maastricht Netherlands; ^4^ Research Department Forensic Psychiatric Centre De Kijvelanden Poortugaal Netherlands

**Keywords:** impulsive behavior, substance use disorders, neurofeedback, craving, offenders

## Abstract

**Background:**

Impulsivity and substance use disorder (SUD) are strongly interconnected, with persons scoring high on impulsivity being more vulnerable to develop substance abuse, facing more challenges for successful treatment, and being more prone to engage in criminal behavior. Studies have shown that impulsivity and craving for substances are strongly correlated. Neurofeedback is an effective treatment to reduce impulsive behavior. This study intends to determine to what extent a neurofeedback-intervention that is aimed at reducing impulsivity can also reduce levels of craving in forensic patients with SUD and comorbid Axis I and/or II diagnoses.

**Objective:**

The main objective of this study is to investigate to what extent a reduction in impulsivity by a sensorimotor rhythm (SMR)-neurofeedback intervention will lead to a reduction in craving in a population of forensic psychiatric patients with a diagnosis of SUD.

**Methods:**

Participants will be male SUD patients with various comorbidities residing in an inpatient forensic treatment facility approached through treatment supervisors for participation. Participants have tested positive for drug use in the past 24 months. The study consists of 2 parts: a randomized controlled trial (RCT) and a n-of-1 clinical series. In the RCT, 50 patients will be randomly assigned to an intervention (n=25) or a control (n=25) condition. Patients in the intervention group will receive 20 SMR neurofeedback sessions aimed at reducing impulsivity; participants in the control group receive treatment-as-usual (TAU). Additionally, 4 in depth n-of-1 clinical trials will be conducted where effects of an SMR neurofeedback intervention will be compared to effects of sham neurofeedback.

**Results:**

Results of this study are expected by the end of 2017.

**Conclusions:**

This protocol describes the design of a study testing the effects of an impulsivity-based neurofeedback protocol among forensic patients with SUD and various comorbidities. We expect a significant reduction in impulsive behavior, level of craving, and actual drug-use for participants receiving the SMR neurofeedback protocol. The n-of-1 approach might help to explain effects possibly found in the RCT study since it allows for a more direct focus on treatment effects by following participants more closely and thereby being able to directly attribute behavioral and neurophysiological change to the SMR neurofeedback protocol employed.

**ClinicalTrial:**

Dutch National Trial Register NTR5386; http://www.trialregister.nl/trialreg/admin/rctview.asp?TC=5386 (Archived by WebCite at http://www.webcitation.org/6nXLQuoLl)

## Introduction

### Background

Impulsivity has been defined as a dysfunctional trait, leading to a tendency for an individual to display behavior that is performed with little or inadequate forethought [1] and might be criminal and possibly harmful to oneself or to others [2]. Impulsivity has been operationalized in different ways with inadequate behavioral inhibition being conceived as one of the key factors [3]. Impulsive behavior is hypothesized to involve a disinhibition of cognitive control that occurs without conscious deliberation [4].

Several studies have demonstrated that substance use disorder (SUD) is strongly associated with elevated impulsivity scores on various measures [3]. In SUD, the use of a substance is continued even though a person is aware of the negative consequences of prolonged drug use. This can be explained by deficient inhibitory control over drug-taking which provides immediate (positive) reinforcement [2]. Higher levels of impulsivity were found in individuals scoring high on alcohol, stimulant, and opiate use [3] as measured by self-report instruments, such as the Barratt Impulsivity Scale (BIS-11) [4]. Individuals with combined cocaine and alcohol abuse show impaired response inhibition as compared to controls on continuous performance tasks measuring impulse control such as the cued go/no-go task [5]. Furthermore, a strong relation between elevated impulsivity scores during childhood/early adulthood and substance abuse problems later in life has been observed, indicating that heightened levels of impulsivity might precede the development of substance abuse problems (in Hawkins et al [6], for example). In alcoholism, for example, behavioral disinhibition as assessed with a novelty-seeking scale has been shown to predict early onset alcoholism [7]. Individuals scoring high on impulsivity are therefore more prone to develop SUD than healthy controls and more often exhibit antisocial behavior [7].

The concept of impulsivity has been of particular interest in studies involving criminal offenders, as these individuals often suffer from major mental disorders and are therefore more likely to be involved in criminal acts than persons without major mental disorders [8]. In criminal offenders, cluster B personality disorders and schizophrenia are frequently diagnosed [9]. Comorbidity rates between these disorders and SUD are as high as 70% [9]. Generally, treatment of SUD has proven to be difficult, with relapse rates as high as 60% after treatment in opiate abusers [10]. For patients with a criminal history and a combination of SUD and comorbid disorders characterized by high levels of impulsivity, long-term treatment outcomes are worse [9]. High impulsivity levels both predict early relapse and increase chances of premature termination of treatment [11]. This, in turn, increases the risk of recidivism in criminal behavior [12]. Adequate treatment for this vulnerable patient population is therefore extremely important, as impulsivity can be understood as an important risk factor in both the onset of SUD as well as post-treatment relapse [13].

### Neurofeedback Treatment for Impulsivity and Substance Use Disorder

Electroencephalographic (EEG) spectral analysis is a frequently used method to compare healthy controls with prolonged drug users by focusing on differences in the (relative) strength of naturally occurring rhythms in the EEG (in Alper et al [14], for example). EEG alterations most commonly found in individuals with SUD are characterized mainly by alterations in the strength of theta (4-8 Hz), alpha (8-12 Hz), and beta (12-20 Hz) frequency bands [15] and are hypothesized to be related to symptoms of drug use disorder, such as over attention to drug cues, feelings of restlessness, and loss of impulse control. Although alterations in several EEG spectral measures have been observed that vary by type of addiction, they persist even after drug abuse is in remission [14].

Neurofeedback is an intervention that uses real-time EEG measurements and displays information about these EEG measurements back to the participant, allowing them to not only see but also change their brain electrical activity over time [16]. By principles of operant conditioning, participants learn to reinforce or inhibit specific frequencies of the EEG activity [17] and thereby normalize abnormal EEG states, which in turn aims at changing abnormal psychological states [18]. Sensors are placed on the scalp and moment-to-moment information about brain activity is fed back to the participant [19].

Several studies have shown neurofeedback to be a promising intervention for various disorders, ranging from SUD to attention deficit hyperactivity disorder (ADHD) [16]. In SUD, a widely used neurofeedback protocol is the Scott-Kaiser modification of the Peniston Protocol, consisting of a combination of sensorimotor rhythm feedback (SMR, 12-15 Hz) followed by alpha-theta based feedback [17]. With this type of protocol, patients first receive neurofeedback that focuses on reinforcing SMR (12-15 Hz) while inhibiting slower frequencies such as delta (2-5 Hz) and theta (5-8 Hz) and also inhibiting high beta (ranging from 18-30 Hz) [15,17]. This type of feedback is first employed for 10 to 20 sessions before the neurofeedback protocol is switched to an alpha-theta based protocol, where alpha (ranging from 8-12 Hz) is decreased while theta (5-8 Hz) is augmented until the amplitude of alpha drops below the level of theta [15,17]. The Scott-Kaiser modification of the Peniston Protocol has shown to be effective in opiate-dependent patients as well as in patients with a mixed substance dependency, as it led to the reduction of feelings of craving [15], a powerful predictor of relapse in drug-taking [20-21] and therefore promoted treatment attendance and abstinence rates of participants [17]. As most criminal offenders with SUD also suffer from comorbid psychiatric conditions however, treatment with neurofeedback may become more complicated [16]. For patients having a combination of impulsivity issues due to comorbidity with other psychiatric disorders, as well as substance abuse problems, it is suggested that an SMR-enhancing neurofeedback protocol should be applied to address the issue of impulsivity first [22]. Studies performing a neurofeedback protocol consisting of suppressing slow waves such as theta (4-7 Hz) and enhancing faster waves such as SMR (12-15 Hz) have demonstrated an improvement of impulse control in a population of students (in Egner and Gruzelier [23], for example) and have shown to positively affect motor control and cortical inhibitory function (in Sokhadze et al [16], for example). This type of neurofeedback protocol is also commonly applied with patients suffering from the hyperactive-impulsive ADHD subtype, and there are many studies reporting reduction in impulsivity after treatment (in Fuchs et al [24], for example). Several studies have shown that impulsivity and craving for substances are strongly correlated no matter the administered drug of choice. For example, in a study by Tziortzis et al [25] with methamphetamine users, individuals with higher levels of impulsivity reported significantly more craving than individuals scoring lower on impulsivity. In alcohol-dependent patients, higher scores of craving were correlated with higher self-reported impulsivity on the BIS-11 [26]. Moeller et al [27] found a significant correlation between the motor impulsivity subscale of the BIS-11 and craving in a population of cocaine-dependent subjects. Also for cocaine-dependent patients, higher impulsivity was associated with greater severity of addiction symptoms such as craving [28,29]. Also, contemporary neuropsychological models stress impulsivity and SUD to be the result of the same imbalance between bottom-up and top-down neural systems [30,31]. Bottom-up systems concern subcortical brain circuitry promoting impulsive reward behavior (regardless of long-term outcomes), whereas top-down processes concern reflective and self-control functions driven by prefrontal brain circuitry [32]. Within SUD, chronic substance abuse may produce neural changes leading to a structural state of disinhibition and impulsivity [33,34], causing immediate reaction to substance-related cues that elicit craving [35]. Not only acute but also prolonged effects of substance abuse have proven to be of great influence in disrupting these neuropsychological mechanisms, therefore maintaining problems with inhibitory control even after drug use is terminated [36]. Although impulsivity and craving are both independently identified as key elements in SUD, to date, there has been no study investigating whether a reduction in one will also lead to a reduction of the other.

### This Study

Although the relationship between impulsivity and symptoms of SUD such as craving and actual drug use has been established, to date there is no evidence about the effects of an impulsivity-based neurofeedback protocol and its effectiveness on impulsivity and on symptoms of SUD. This study aims to examine the treatability of impulsivity with an SMR-neurofeedback intervention in a population of forensic psychiatric patients with SUD and comorbid Axis I and/or II disorders. It also aims to investigate whether a reduction of impulsivity through an SMR-based neurofeedback protocol will also result in a reduction of SUD symptoms such as craving and actual drug use.

The study will combine a randomized controlled trial (RCT) design with an n-of-1 clinical trial. The RCT allows for investigating to what extent an SMR-neurofeedback protocol can reduce craving and actual drug use by augmenting levels of impulsivity for forensic psychiatric patients at a group level. However, RCTs have several disadvantages. First, they focus on between-group differences, making it difficult to determine the exact working mechanisms of neurofeedback at the single patient level. Despite the fact that the number of studies employing neurofeedback has increased over the past 2 decades, to date the underlying working mechanisms of neurofeedback remain unclear. Success of treatment is usually determined by a reduction in subjective complaints or based on other behavioral measures, independent of patients’ responses to neurofeedback on a neurophysiological level (eg, change in mean amplitude of brain frequencies). Second, most RCTs focus on participants with single, well-defined disorders or diagnoses, making it difficult to apply previous findings to patients who have a more complex psychopathology as is usually the case in forensic patients. Third, finding a reduction in subjective complaints could partially be explained by the interaction with the person giving the treatment, as this occurs with almost all frequently given types of therapy in the psychological field [37]. To rule this out, large RCTs with a treatment and a sham arm are necessary. Unfortunately, these studies are very difficult to conduct in a forensic psychiatric setting due to the fact that forensic patients generally have low levels of treatment compliance [38]. As the current study concerns a single-site study with only a limited number of patients who fit the inclusion criteria to begin with (but on forehand sufficient according to power analysis), adding a sham arm to the RCT would most likely further reduce the motivation of patients to participate and hence increase nonresponse. However, insight into possible sham effects is needed to differentiate between specific and nonspecific treatment effects which are independent of the neurofeedback trainer. Finally, RCT studies showing treatment effects of neurofeedback often vary in the applied protocols, number of sessions, and treatment intensity. To date, there have been no guidelines developed that specify these neurofeedback parameters. Especially for forensic patients, developing a treatment that is well applicable and helps to reduce symptoms of SUD is of great importance, as forensic treatment is aimed at protecting society and reducing the risk of reoffending. By adding several n-of-1 clinical trials we attempt to cope with these disadvantages. A well-conducted n-of-1 trial allows testing of the specific working mechanisms of neurofeedback in a single patient and is therefore able to detect detailed behavioral and neurophysiological changes that can then be attributed more definitely to neurofeedback treatment.

### Objectives

Primary outcome variables are the degree of impulsivity as measured with the Dutch version of the BIS-11 [39]; inhibitory control as measured with a cued go/no-go reaction time task [40]; degree of drug craving as measured with an altered version of the Desire for Alcohol Questionnaire (DAQ) [41]; actual drug use as measured with urine, saliva, or breathalyzer analysis; and changes in resting state EEG pattern.

Primary objective: To what extent does a reduction in impulsivity by using SMR-neurofeedback result in a reduction of core symptoms of SUD such as craving and actual drug use in a population of forensic psychiatric patients with a diagnosis of SUD?

Secondary objectives:

1. To what extent can an SMR-based neurofeedback intervention reduce levels of impulsivity as measured by BIS-11 and a cued go/no-go task in a population of forensic psychiatric patients with a diagnosis of SUD?

2. To what extent can an SMR-based neurofeedback intervention reduce levels of craving as measured by self-report questionnaire DAQ-SF (short form) in a population of forensic psychiatric patients with a diagnosis of SUD?

3. To what extent can an SMR-based neurofeedback intervention reduce actual drug use as measured with urine, saliva, or breathalyzer analysis in a population of forensic psychiatric patients diagnosed with SUD?

## Methods

### Overview

This study will be conducted according to the principles of the Declaration of Helsinki (version 59, Seoul, October 2008) and in accordance with the Medical Research Involving Human Subjects Act. It has been approved by the medical ethical council of Brabant, the Netherlands (study number NL46390.008.13).

This study takes place in Forensic Psychiatric Centre (FPC) Dr S van Mesdag, a maximum security inpatient forensic treatment facility in Groningen, the Netherlands. Patients in this treatment facility are male criminal offenders with at least one Axis I or II diagnosis and considered to be at risk for criminal recidivism if not treated properly. About 70% of all patients treated in this facility have a comorbid diagnosis of SUD [9].

### Randomized Controlled Trial

A randomized controlled trial with N=50, where 25 participants are randomly assigned to treatment as usual (TAU) combined with 20 SMR-based neurofeedback sessions and 25 participants are randomly assigned to TAU only, without neurofeedback intervention. The 2 groups are compared pretreatment (T0) and posttreatment (T1) on variables linked to the research questions. Both groups will receive pre- and posttreatment measurements with an interval between T0 and T1 of approximately 10 weeks in which participants in the intervention group will receive 20 neurofeedback treatment sessions and participants in the control condition will follow TAU.

The design of this part of the study is a 2×2 design with the condition (neurofeedback vs TAU) as a between-subjects factor and time as a within-subjects factor (pre- and postintervention).

### N-of-1 Clinical Trial

To zoom in on specific treatment effects, 4 single case studies with an A^1^B^1^A^2^B^2^design (single time series) will be conducted, of which 2 single case studies will apply an actual SMR-neurofeedback protocol and 2 single case studies will apply a sham neurofeedback training. The clinical trial will be single-blinded, indicating that participants do not know which part of the training they will receive. Participants are selected from the control group of the previously described RCT protocol who have already completed pre- (T0) and posttreatment (T1) measurements. Inclusion in the n-of-1 trial will be selective: participants with the highest scores on outcome measures on T1 of the RCT will be approached first as it is believed that these patients have the highest need for treatment. However, allocation to treatment (sham or real) will be random.

For a detailed description of this design of n-of-1 studies, see Rizvi and Nock [42]. Basically, in this design, a baseline period (A^1^: no treatment, lasting 3 weeks) is followed by a treatment period (B^1^: neurofeedback, sham or real, lasting 4 weeks and resulting in 8 neurofeedback sessions), which is followed by a period where treatment is withdrawn (A^2^: lasting 3 weeks). During all periods, outcome measures DAQ-SF and BIS-11 will be assessed 2 times per week. At the end of the A^2^period, statistical analyses are applied to test for significant improvements in study end points. In cases of significant improvement during treatment, a second period of neurofeedback, B^2^(sham or real), will be applied. This way, if neurofeedback does not prove to be effective within B^1^, participants will not be burdened with the requirement of completing more sessions. It is expected that patients who have not shown any significant improvement during neurofeedback sessions in B^1^will not show any further improvements when undergoing more sessions. After completion of the study, patients and treatment supervisors will be debriefed about whether the neurofeedback intervention was real or sham.

To test for transient effects of the neurofeedback intervention, a follow-up measurement of resting state EEG, BIS-11, DAQ-SF, and cued go/no-go task will be performed 12 months after completing the posttreatment measures for both participants in the intervention group of the RCT and for participants in the n-of-1 clinical trial.

### Participants

A power analysis calculation for the RCT using G*Power 3 (Department of Psychology) based on a 1-tailed alpha value of .05, a power value of 0.80, and an effect size (*f*) of 0.80 yielded a recommended sample size of 21 participants each in the control and intervention conditions. Given the special research population we aim to select 25 participants for each condition.

Participants are male patients diagnosed with SUD (substance dependency or substance abuse) according to the *Diagnostic and Statistical Manual of Mental Disorders*, Fourth Edition, Text Review (DSM-IV-TR [43]) and currently staying at the treatment facility. Participants have tested positive for drug use in the past 24 months at time of inclusion. Drug use is operationalized as urine, saliva, or breathalyzer analysis testing positive for either marijuana and/or psychostimulant/opioid drugs and/or alcohol. Corresponding with treatment facility policy, nonprescribed medication that is used for recreational drug consumption such as inhaled methylphenidate will also be scored as positive drug testing, as will refusal to undergo drug testing.

Participants are allowed to continue using prescription medication (as prescribed by a psychiatrist or general physician of the treatment facility) but are required to inform researchers of any medication they are currently using or any change in medication during treatment with neurofeedback.

### Recruitment

Recruitment will start with the selection of patients for the RCT part of the study. Participants are approached through treatment supervisors for participation. Treatment supervisors are informed about the general inclusion criteria for this study. Out of all participants that meet the requirements, a random sample of 50 will be drawn and randomly assigned to 1 of the 2 conditions (intervention and control). Prior to participation in the trial all participants are asked to provide written consent. If at this point a participant chooses not to participate in the trial, this will be coded as a nonresponse. Missing numbers of participants will be complemented by randomized allocation of other suitable participants who are willing to participate in order to guarantee the sample size. Once all patients for the RCT have been recruited, recruitment for the n-of-1 clinical trial will begin. All participants will receive a financial reward after completing pre- and posttreatment measurements.

### Measures

#### Electroencephalography

Participants will undergo a 21-channel EEG measurement with Nexus-32 hardware and Biotrace+ software (Mind Media BV). The EEG will be collected from 19 standard 10/20 positions [44] and the right and left mastoid with a sampling rate of 512 samples per second. The left mastoid will serve as the online reference. Flat type electrodes will be placed above and below the left eye and at the outer canthi of each eye to correct for vertical and horizontal eye movements. Participants will be seated comfortably while 5 minutes of eyes closed resting state EEG data is collected. EEG measures will be conducted at T0 and T1 as well as at 12 months follow-up for participants in the intervention group (T2).

For participants in the neurofeedback group, a 1-minute baseline recording over 3 conditions will be conducted before start of the first neurofeedback session and after the last session. EEG signal will be recorded from electrode position Cz against a right ear mastoid reference across the conditions (1) eyes open, (2) eyes closed, and (3) cognitive task (where participants are instructed to solve simple mathematical calculations). These measurements will be used to determine neurofeedback threshold values and to assess change in mean magnitude of frequency bands before and after neurofeedback training.

#### Barratt Impulsivity Scale–11

The Dutch version of the BIS-11 (eleventh edition) [45] is a self-report questionnaire designed to measure the behavioral and personality construct of impulsivity across 3 second-order factors: attentional, motor, and nonplanning. It consists of 30 items scored on a 4-point scale ranging from rarely/never to almost always/always. The BIS-11 has been proven to be an internally consistent measure of impulsivity among inmate populations [45].

#### Cued Go/No-Go Task

The cued go/no-go task is a continuous performance test measuring impulse control by the ability to inhibit prepotent responses [40]. Participants are instructed to respond to a green square by pressing a button as quickly as possible while not responding to a blue square. A go or no-go cue is given before the actual target appears, providing information about the likelihood of an actual go or no-go target [40]. The likelihood of a correct target after a cue is manipulated with a 80/20 ratio, with 80% being a correct cue and 20% being an incorrect cue. Cues are presented with 4 fixed stimulus onset asynchronies (100, 200, 300, and 400 ms), giving participants time to prepare for responding. The cued go/no-go task has been proven to be a useful measurement of impulse control in substance abusing populations [40]. It consists of 250 trials spread over 5 rounds with a 30-second break between each round, taking approximately 20 minutes to complete. Outcome measurements are omission (the participant does not respond when he should respond) and commission errors (the participant responds when he should not respond) and reaction time.

#### Modified Desire for Alcohol Questionnaire

The DAQ-SF [46] is a self-report questionnaire assessing the desire to use drugs at the moment of assessment. It is derived from the original desire for alcohol questionnaire (DAQ) with 36 items. The short-form version of the DAQ consists of 14 item that can be scored on a scale from 1 to 7 ranging from strongly disagree to strongly agree. It consists of 3 factors: (1) strong desires/intention to drink, (2) negative reinforcement, and (3) ability to control drinking. The abbreviated version has been shown to be reliable in measuring alcohol craving [46].

All questions of the original questionnaire are designed to measure craving purely for alcohol; however, within the treatment facility alcohol use is less common than other drug use (such as marijuana and/or cocaine). Therefore, questions from the questionnaire have been altered so they can fit any type of drug dependency. An example of this is “My desire to drink seems overpowering” which has been altered to “My desire to use drugs seems overpowering.”

#### Instrument for Forensic Treatment Evaluation

The Instrument for Forensic Treatment Evaluation (IFTE) is an observational treatment evaluation instrument consisting of 22 items measuring 3 factors: Problematic behavior, protective behavior, and resocialization skills. It is scored on a 17-point Likert scale with 5 anchor points: none, rarely, sometimes, often, and always [47]. The IFTE assesses forensic risk behaviors such as impulsivity, hostility, and violating treatment conditions. These risk behaviors might be manifestations of impulsive behavior and could help assess engagement in impulsive behavior that is not assessed by the BIS-11 and the cued go/no-go task. Furthermore, the IFTE also assesses cooperation with treatment, which measures the amount of effort a patient puts in to make progress in his treatment, giving an indication of the degree of commitment (and thereby, motivation) of a patient to forensic treatment. The IFTE is scored twice a year by clinicians involved in patients’ treatment as part of routine outcome measurement within the treatment facility. Patients also score the IFTE on a self-report version of the original IFTE (IFTE-SR), where they can give an indication of treatment progress during the past 6 months. Scores of the IFTE and IFTE-SR are assessed from the moment a patient arrives at the treatment facility up until release. Therefore, scores on the IFTE are available throughout the research. Relevant scores included in this study will be assessments 6 months prior to inclusion up until 12 months after the last measurement.

#### Actual Drug Use

Drug testing is performed on a regular basis, usually once every 2 weeks. Whenever staff suspects illegal use of substances within 2 moments of drug testing, spontaneous and unexpected drug testing can be performed. Number of drug tests will be counted, as will be positive (meaning drug use in the period of time since last drug test) and negative (meaning no drug use since last testing) outcome scores. Drug testing is done in the form of urine, saliva, or breathalyzer (for alcohol use only) analysis.

#### Covariates

Covariates are sociodemographic characteristics; specific psychopathology; duration of forensic treatment; actual drug use during the past 24 months (or as long as patients reside in the treatment facility); medication use; clinical risk assessment score (Historical/Clinical/Future-Revised, HKT-R) [48]; actual drug use; and mean score of delta, theta, alpha, beta, and gamma resting state EEG frequency band power. Covariates will be collected through case file information. Medication and medication change will be categorized according to class of medication (eg, benzodiazepines, antipsychotic medication).

### Intervention

All participants already receive TAU at the moment of inclusion. They will continue to do so during the course of this trial. Type of TAU is dependent on disorder and behavior but can range from cognitive behavioral therapy, psychotherapy, and psychomotor therapy to relapse prevention treatment and can be either individual treatment or in-group treatment. Treatment can also be supplemented by medication for psychotic symptoms or depressive symptoms, for example. In some rare cases, aversion or craving reducing medication is prescribed.

Participants in the intervention condition of the RCT will receive 20 neurofeedback sessions, each lasting approximately 40 minutes. EEG magnitude is measured across delta (0.5-3.5 Hz), theta (3.5-7.5 Hz), alpha (7.5-12 Hz), beta (12-20 Hz), SMR (12-15 Hz), high beta (20-32 Hz), and gamma (32-49 Hz) frequency bands. To reduce inattention and impulsivity, a conventional neurofeedback protocol will be used that consists of suppressing theta magnitude and enhancing SMR magnitude [49,50]. The aims of the neurofeedback sessions are therefore to reduce slow waves (specifically theta, 3.5-7.5 Hz, and if necessary delta, 0.5-3.5 Hz) and increase faster waves (SMR, 12-15 Hz). A maximum of 3 different frequency bands will be trained during each session. Neurofeedback training will be performed on the EEG signal recorded from electrode position Cz against a right ear mastoid reference.

For the n-of-1 design of the trial, 2 participants will receive the SMR-neurofeedback intervention and 2 participants will receive sham neurofeedback.

Real and sham neurofeedback procedures will be similar (eg, electrode position, preparation, instructions given to participants) except that for the sham neurofeedback training group, participants are instructed to enhance an irrelevant frequency band that is randomly chosen from higher beta bands (20-23 Hz, 23-26 Hz, 26-29 Hz, and 29-32 Hz). Therefore, no specific frequency band is systematically modulated and thus should not result in desired treatment outcomes. Participants will still be given positive feedback and be able to influence the video games in order to minimize possible irritation of participants.

Neurofeedback will be applied as implemented within the BrainMarker software engine (BrainMarker Device, Brainmarker BV Gulpen).

Participants will be shown simple video games implemented in the software that will provide feedback about their brain activity. During the video games, they are instructed to be attentive to the feedback (no movement/movement of objects) in the video game and to find the most successful strategy to reach the goal of the game. Example of such video games are a car moving on a road, where participants are instructed to keep the car in the right lane of the road, or a basketball court where participants are instructed to try to throw the ball in the basket. The video game–based neurofeedback rounds will last 1 minute at a time, with a short break between rounds. Also, movie-based neurofeedback will be applied. During movie-based neurofeedback participants will watch a digital video disk of their own choice and be instructed to keep the monitor as free as possible from black curtains appearing on both sides of the monitor and keep the volume of the movie at an audible level. Movie-based training will last 90 seconds at a time with a short break when necessary. Participants will receive both game- and movie-based neurofeedback in each session.

Thresholds will be set manually in a way that if a participant maintains the reinforced frequency band above a threshold for 80% of time, positive feedback will be received. To determine threshold values, mean magnitude of the baseline measurement across the 3 conditions described above will be used to roughly assess threshold values for the neurofeedback training. For each training session, mean magnitude values will be calculated for all frequencies.

### Statistical Analysis

All statistical analysis will be conducted using SPSS version 19 (IBM Corp). Summarizing descriptive statistics and frequency tables will be provided.

#### Randomized Controlled Trial

Resting state EEG data will be analyzed using custom-made Matlab R2012b scripts [51]. A repeated measures multivariate analysis of variance with factors condition (neurofeedback vs control) and frequency band (delta, theta, alpha, beta, or gamma) will be conducted. If main or interaction effects are observed, post hoc tests will be used to determine which levels of the factors are explaining the observed effects.

Repeated measurement with time (pre- [T0] and postintervention [T1]) as the within-subject factor and group (control vs intervention) as the between-subject factor will be conducted for the DAQ-SF, BIS-11, IFTE, and IFTE-SR. If main or interaction effects are observed, post hoc test will be used to determine which levels of the factors are explaining the observed effects. A repeated measures analysis of covariance will be conducted to examine differences in actual drug use as dependent variables to test for a moderating effect of impulsivity on craving and actual drug use.

#### N-of-1 Trial

First, a time-plot will be inspected using the autocorrelation coefficient (ie, correlogram) [52]. After inspection, time-series analysis will be applied to test for significant slope and level changes as well as a trend analysis. Analysis techniques will be based on the study by Solanas et al [53].

## Results

Results of all measurements will be expected by the end of 2017 and will be published in corresponding articles.

## Discussion

This study aims to evaluate the efficacy of an SMR-based neurofeedback treatment on reducing impulsivity in a population of inpatient forensic patients. Possible effects of a reduction in impulsivity on substance abuse will be assessed as well. We expect a significant reduction in impulsive behavior, level of craving, and actual drug use for participants receiving the SMR-neurofeedback protocol. The n-of-1 approach might help to explain effects possibly found in the RCT study since it allows for a more direct focus on treatment effects by following participants closely and thereby being able to directly attribute behavioral and neurophysiological change to the SMR-neurofeedback protocol employed. The study aims to extend previous findings on the efficacy of neurofeedback treatment in reducing impulsivity, not only by linking possible findings regarding a reduction of impulsivity to substance abuse symptoms but also by examining effects in a forensic psychiatric population with various comorbid disorders.

Studies about the efficacy of neurofeedback in a psychiatric forensic setting, in which the population is characterized by various comorbidities and various kinds of medication, are lacking. In our study, exclusion criteria are kept to a minimum to include as many participants with SUD as possible and to be able to generalize effects of an SMR-neurofeedback treatment over different types of comorbidities.

Although RCTs with a treatment and a sham treatment arm are considered the gold standard in research, conducting large trials is often times difficult in forensic settings; treatment motivation might be low for the type of patients in the treatment facility because they are placed under compulsory inpatient custody and are not seeking treatment due to inner motivation for change. In RCTs, number of participants usually has to be quite high to reach the desired effect size [54]. Participating patients might be even less inclined to take part in the trial if they know that they might end up in a placebo condition.

By employing an n-of-1 approach combined with an RCT, this study might help shed light on the underlying mechanisms of neurofeedback because an n-of-1 approach allows closer monitoring of treatment effects and provides valuable insight into an individual’s treatment progress that might otherwise be lost in a between-group design [42].

If effective, neurofeedback could be a noninvasive treatment option for the reduction of impulsivity, which may lead to a reduction in feelings of drug craving and in actual drug use. Both impulsivity and drug-seeking behavior are known to hamper treatment progress and are strongly linked to criminal behavior [32]. By reducing impulsivity, chances of successful treatment for SUD may increase, thereby decreasing the risk for relapse in drug use and reducing criminal behavior.

There are several important issues to consider that might influence the results. First of all, participants are not selected based on their level of impulsivity. Even though the most commonly observed disorders in the treatment facility are schizophrenia and personality disorder and both types of disorders are associated with increased impulsive behavior, not all suitable participants might show elevated levels of impulsivity. Studies have shown that although there is evidence that heightened impulsivity can be found across different types of substance use disorders, there is still substantial heterogeneity on impulsivity levels within these groups [2]. A recent study by Albein-Urios et al [55] found several subgroups of addicted individuals that exhibited different clinical presentation and most interesting, different severity levels of craving. In the study, a latent class analysis showed that greater impulsivity levels were associated with worse clinical outcomes, whereas conventional diagnostic groups showed no significant differences on outcome variables. Also, there have been studies that show that antisociality is actually associated with better impulse control, independent of extent of drug use [56]. To ensure a sufficient number of participants, inclusion criteria in this study are quite lenient, which may provide heterogeneity within this sample. Ideally, participants would have to present with the same diagnoses, same type of medication, etc, however, this would limit the number of available participants to such an extent that it will be hard to find any effects. The heterogeneity of the population makes it possible that an impulsivity-based neurofeedback protocol might not result in a reduction of craving and actual drug use.

Also, participants will be included who have tested positive for drug use in the past 24 months. This implies that there will also be participants whose substance use disorder is in early remission. Although substance abuse–related symptoms such as craving are known to persist even after drug use is terminated, this period of time might be too long for these participants to report any craving at the moment of the administered questionnaire.

## Appendix

Multimedia Appendix 1 CONSORT-EHEALTH checklist V1.6.1 [57].

## Abbreviations

ADHD: attention deficit hyperactivty disorder
BIS-11: Barratt Impulsiveness Scale
DAQ: Desire for Alcohol Questionnaire
DAQ-SF: Desire for Alcohol Questionnaire–Short Form
DSM-IV-TR: Diagnostic and Statistical Manual of Mental Disorders, Fourth Edition, Text Review
EEG: electroencephalograph
FPC: Forensic Psychiatric Centre
HKT-R: Historical/Clinical/Future–Revised
IFTE: Instrument for Forensic Treatment Evaluation
IFTE-SR: Instrument for Forensic Treatment Evaluation–Self-Report
NFB: neurofeedback
RCT: randomized controlled trial
SMR: sensorimotor rhythm
SUD: substance use disorder
TAU: treatment as usual
